# Sonic-assisted antibacterial photodynamic therapy: a strategy for enhancing lateral canal disinfection

**DOI:** 10.1186/s12903-023-03801-6

**Published:** 2024-01-02

**Authors:** Yanhuang Wang, Lishan Lei, Jing Huang, Zhiyu Cai, Xiaojing Huang

**Affiliations:** 1 Fujian Key Laboratory of Oral Diseases & Fujian Provincial Engineering Research Center of Oral Biomaterial & Stomatological Key laboratory of Fujian College and University, School and Hospital of Stomatology, Fujian Medical University, Fuzhou, 350002 PR China; 2 Department of Stomatology, Fujian Medical University Union Hospital, Fuzhou, 350002 PR China

**Keywords:** Antimicrobial photodynamic therapy, Methylene blue, *Enterococcus faecalis*, Lateral canal, Sonic

## Abstract

**Background:**

Bacterial infections in lateral canals pose challenges for root canal treatment. This in vitro study aims to evaluate the antibacterial efficacy of sonic-assisted methylene blue mediated antimicrobial photodynamic therapy (MB-aPDT) against *Enterococcus faecalis* (*E. faecalis*) in infected lateral canals.

**Methods:**

Sixty-five premolars infected with *E. faecalis* in lateral canals were randomly divided into five groups (n = 13) and treated with : (1) 5.25% NaOCl (positive control); (2) Saline (negative control); (3) Sonic-assisted MB-aPDT; (4) 3% NaOCl + MB-aPDT; (5) 3% NaOCl + sonic-assisted MB-aPDT, respectively. The antibacterial efficacy was evaluated by the colony- counting method (CCM) and scanning electronic microscope (SEM).

**Results:**

Both 5.25% NaOCl and the 3% NaOCl + sonic-assisted MB-aPDT exhibited the most effective while comparable antibacterial effects without significant statistical difference (P > 0.05). Furthermore, the antibacterial effect of the 3% NaOCl + MB-aPDT group was significantly higher compared to that of the sonic-assisted MB-aPDT group (P < 0.05). The SEM results demonstrated notable morphological alterations in *E. faecalis* across all experimental groups, except for the negative control group.

**Conclusion:**

The concentration of NaOCl can be reduced to a safe level while preserving its antibacterial efficacy through the synergism with the sonic-assisted MB-aPDT in this study.

## Background

The presence and effects of microorganisms and their byproducts play pivotal roles in the development and advancement of endodontic and periapical diseases [1, 2]. Recent studies indicate that the enhanced rates of recuperation in the periapical area are achieved when the microbial load diminished through root canal therapy [3]. Therefore, the effective elimination of bacteria from infected canals stands as one of the primary goals of in endodontic treatment. Nevertheless, in multiple cases, bacteria persist within dentin tubules, lateral canals and other anatomic complexities, evading mechanical preparation and chemical disinfection, thereby leading to resistance [4].

Lateral canals, commonly observed in permanent human teeth, establish a connection between the root canal and the periodontal area, serving as a potential pathway for bacterial dissemination from the pulp to the periodontal tissues [5]. The employment of mechanical techniques for root canal preparation renders instruments incapable of accessing the lateral canals, increasing the challenge of infection control and potentially leading to the failure of root canal treatment [6]. Consequently, the effective elimination of bacteria from the lateral canals exerts a substantial impact on the treatment outcomes.

*Enterococcus faecalis* (*E. faecalis*), a Gram-positive (G^+^) facultative anaerobe, demonstrates the ability of forming biofilms [7, 8]. This microorganism exhibits the capacity of penetrating various irregular structures within the root canal system, including dentin tubules and lateral canals, and exhibits resilience under conditions of nutrient deprivation and high alkalinity [7, 9]. Furthermore, this microorganism plays an essential role in the persistence of periapical inflammation that is resistant to treatment [10].

Sodium hypochlorite (NaOCl) is commonly used as an irrigation solution in clinical settings [11, 12]. However, its antimicrobial efficacy and toxicity are proportional to its concentration [13]. Despite efficient antimicrobial agent in endodontic treatments, it is important to recognize its potential shortcomings. The most important one is the necessity for high concentrations to achieve sufficient antibacterial efficacy, which consistently, elevates the toxicity, including chemical burns and necrosis [14, 15]. These adverse reactions may cause discomfort and the prolongation of the healing process. Furthermore, NaOCl has been certified to exhibit a high surface tension, which limits its capacity to penetrate irregular areas like the lateral canals [16]. Over the past few years, a new approach to fighting microorganisms, known as antimicrobial photodynamic therapy (aPDT), has emerged as a valuable addition in root canal disinfection [17–21]. This technique involves the use of a light source and a photosensitizer in an aerobic environment to induce bacterial lysis and death via the generation of reactive oxygen species (ROS) [22].

Photosensitizers are essential components of aPDT. In the application of aPDT in root canal disinfection, photosensitizers are typically administered into the canal through a needle and syringe, resulting in limited permeability [23, 24]. Researches indicate that the employment of ultrasonic waves to activate the photosensitizer in aPDT improves its permeability and enhances its effectiveness against bacteria [25]. However, the rigid metal tip of the ultrasonic instrument has the potential for causing mechanical damages upon contact with the dentin wall and may pose challenges in reaching the apical region, especially in curved root canals [26].

The EDDY device, powered by sonic technology, can produce a three-dimensional (3D) movement within the root canal, creating “cavitation” and “acoustic” streaming effects similar to those generated by ultrasonic devices [27]. Unlike ultrasonic devices with metallic tip, the EDDY device utilizes a flexible polyamide tip to overcome the limitations associated with the former [28]. An earlier research has demonstrated that sonic-assisted treatment can enhance the penetration of photosensitizers [29]. However, the antibacterial effectiveness of this approach remains unexplored.

Thus far, scant information exists regarding the efficacy of aPDT against bacteria in lateral canals, especially when combined with sonic-assisted treatment. Therefore, the purpose of this study was to evaluate the efficacy of sonic-assisted methylene blue mediated aPDT (MB-aPDT) in eliminating *E. faecalis* in infected lateral canals by comparing that of NaOCl, a gold standard endodontic irrigant. This study aims to explore a new strategy for the effective elimination of bacteria in lateral canals, while consequently reducing the concentration of NaOCl. The ultimate goal of this study is to promote the success rates of root canal treatment.

## Methods

### Tooth species

This study selected sixty-eight fully developed premolars with straight and single root canals that were extracted from adults aged between 18 and 30 years for orthodontic purposes. Approval for this in vitro study was granted by the ethics committee of School and Hospital of Stomatology, Fujian Medical University, Fujian Stomatological Hospital (No: 2021.58).

### Sample preparation

After the initiation of the pulp chamber, the length of the root canal was determined using a 15#K file before proceeding to root canal preparation with a Reciproc file (VDW, Munich, Germany). All teeth were prepared to a size 40 #. During the preparation procedure, 2.5% NaOCl solution was used for root canal irrigation, which was then neutralized with 2 mL of a 5% sodium thiosulfate solution. Subsequently, 17% EDTA solution was irrigated in the canals for one minute, followed by saline rinses.

### Artificial lateral canals

The method proposed by *Venturi et al.* was used to prepare the lateral canals [30]. All samples were decalcified with 5% nitric acid, and the liquid was changed every 24 h. After the decalcification process, the teeth underwent a 24-hour rinse with tap water to remove any remaining nitric acid residue. Subsequently, a sterile 40 # gutta-percha point was inserted into the primary root canal, while two 10 C + files were separately placed 3 mm away from the working length to create the lateral canals. These files were positioned perpendicular to the external surface on both the buccal and lingual walls. Following the completion of lateral canals preparation, the samples were immersed in methyl salicylate for one week to restore their hardness. Then, all specimens underwent sterilization using ethylene oxide. Each specimen was transferred to an Eppendorf tube containing BHI broth and placed in an incubator at 37 °C for 24 h to confirm sterility. The turbidity of the media was assessed to confirm the absence of bacterial growth.

### Bacterial inoculation and lateral canals contamination

*E. faecalis* (ATCC 29,212) in BHI broth was incubated at 37 °C in an aerobic environment and yielded approximately 1 × 10^8^ colony forming units per milliliter (CFU/mL) after 6 h. Each microtube containing a sample was filled with 1 mL of the inoculum, and four cycles of centrifugation were performed following the methodology described by Andrade et al.’s as a reference [31]. To promote biofilm development, the samples were placed in aerobic conditions at a temperature of 37 °C for two weeks with fresh culture media changed daily. Three randomly selected specimens were observed with SEM to confirm the successful establishment of the infection model. Subsequently, the remaining 65 samples were used for conducting antibacterial investigations.

### Photosensitizers preparation

In this study, the photosensitizer used was MB obtained from Sigma in St. Louis, MO. The solution was prepared with a phosphate buffered saline (PBS) concentration of 0.01%.

### Sample size calculation

The PASS 15 software was used to determine the sample size, resulting in a minimum of eight participants in each group. This was achieved with an alpha error of 0.05, a beta-power of 0.8, and an average effect size of 0.9. To account for any ambiguity in this research, the number of participants in each group was raised to 13.

### Disinfection procedures

The sixty-five specimens were randomly divided into two control groups and three experimental groups, with each group consisted of thirteen samples.

Control groups: The lateral canals in the positive control group underwent treatment with 5.25% NaOCl solutions for 5 min at a flow rate of 0.1 mL/s. In contrast, the negative control group received irrigation with normal saline (NS) solutions for the same duration and flow rate.

Sonic-assisted MB-aPDT: Species in this group were injected with MB solution at a flow rate of 0.1 mL/s. Afterwards, the EDDY device (VDW, Munich, Germany) was used to administer sonic treatment. The tip of EDDY device was positioned 2 mm from the working length and treated for 1 min at a frequency of 6000 Hz. Following this, the specimens were exposed to irradiation from a diode laser device called PeriowaveTM (Ondine Biopharma corporation, Canada). This device emitted light with a wavelength of 660 ± 10 nm and a maximum power output of 150mW. The irradiation lasted for 1 min.

3% NaOCl + MB-aPDT: The lateral canals were treated with a 3% NaOCl solution at a flow rate of 0.1 mL/s. Following a 1-minute irrigation period, 2 mL of 5% sodium thiosulfate was used to neutralize any remaining NaOCl solution. After drying the root canal with sterile paper tips, all specimens in this group were injected and fully immersed in an MB solution for 1 min. The light was then administered using the aforementioned technique.

3% NaOCl + sonic-assisted MB-aPDT: The lateral canals in this group were treated with a 3% NaOCl solution and sonic-assisted MB-aPDT, following the same technique as described above.

### Microbiological analysis

Ten specimens from each group were randomly selected for the sampling process before and after the experiment, respectively, as follows:

First sampling (S1): To determine the initial count of living microorganisms, the dentin debris in the buccal lateral canals of each group was removed using a 15 # K file. The debris was then transferred to sterile Eppendorf tubes containing 1 mL of sterile PBS and agitated for 30 s. After a ten-fold sequential dilution in PBS, 20 µL samples were placed on BHI agar plates and incubate at 37 °C in aerobic conditions for 48 h. Then, count the number of colonies (CFU/mL).

Second sampling (S2): After the treatment, a 15 # K file was used to remove dentin debris from the lingual surfaces of each group. Then, employing the aforementioned method to obtain the residual bacteria.

The antibacterial rate of each group was calculated by the following formula:

$${\text{Antibacterial\,rate}}\,(\%) = ({\text{S}}1-{\text{S}}2) / {\text{S}}1\times100\%$$

### SEM examination

SEM observation was conducted on three randomly chosen specimens from each group without any sampling treatment. The samples were split in half along the axis of the tooth using a chisel to fully expose the longitudinal section of the lateral canals. Following a 24-hour fixation in 2.5% glutaraldehyde, the sections underwent dehydration and were subsequently coated with gold using the sputter-coating technique. Subsequently, a SEM examination was conducted.

### Statistical analysis

The statistical analysis was conducted utilizing SPSS 26.0 software (SPSS Inc, Chicago, IL). The CFU data underwent logarithmic transformation and were assessed using one-way ANOVA and LSD-t test. A level of statistical significance was deemed acceptable when P < 0.05.

## Results

### Establishment of *E. faecalis*-infected lateral canal model

**Fig. 1:**
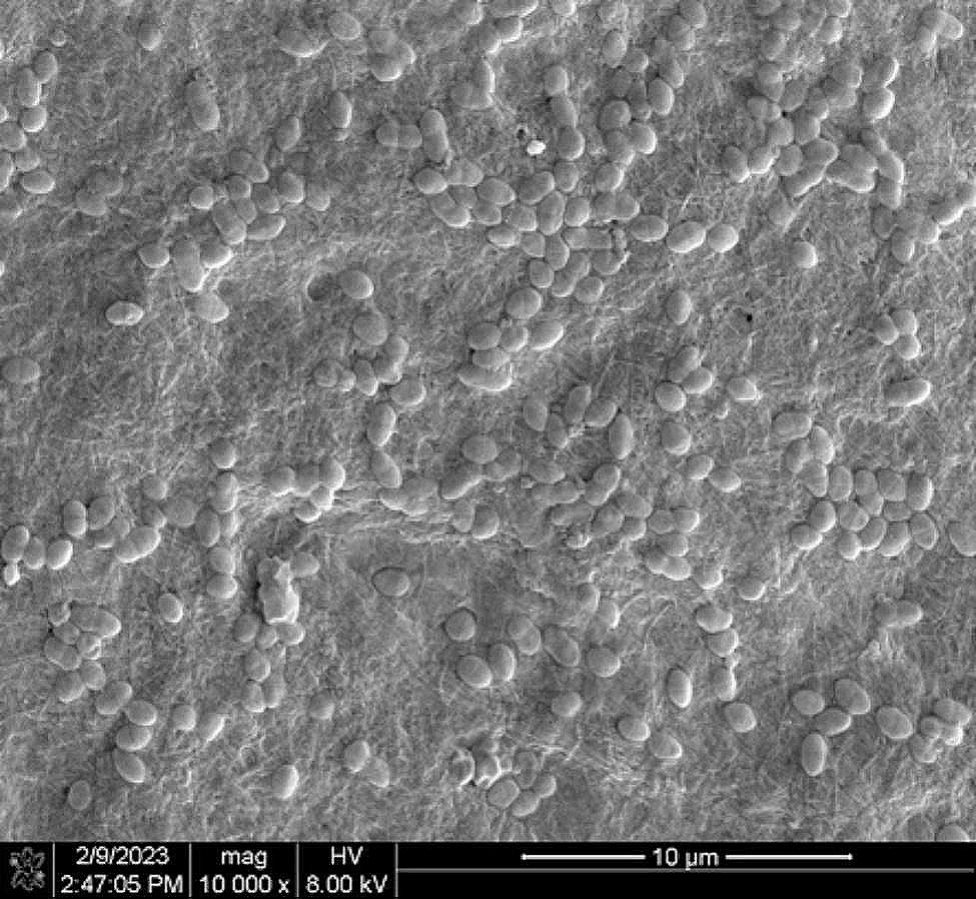
The *E. faecalis* demonstrated a dense colonization in the lateral canals ( × 10,000 )

After a 14-day incubation period, the development of a lateral canal infection model was successfully established, which was firmly supported by the SEM analysis (Fig. 1).

### Antibacterial results

#### Antibacterial effects induced by various treatments

**Fig. 2:**
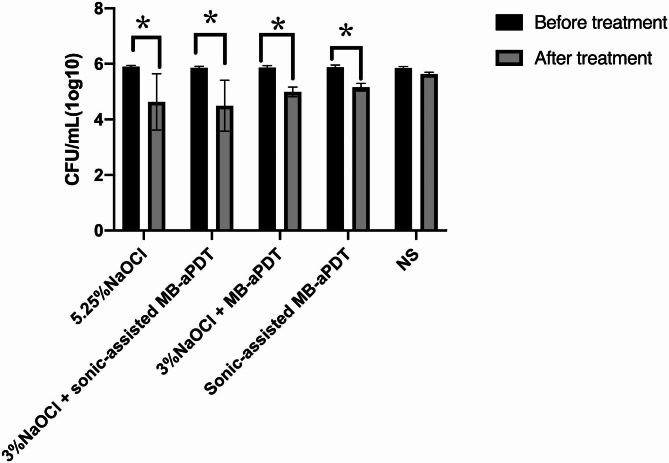
Antibacterial effects induced by various methods. *indicated a significant statistical distinction (*P* < 0.05)

Figure 2 illustrates the findings. All experimental groups, except for the negative control group, exhibited a substantial reduction in bacterial count after treatment, with statistically significant differences observed between the treated and untreated groups (P < 0.05).

#### Comparison of antibacterial rates among groups

**Fig. 3:**
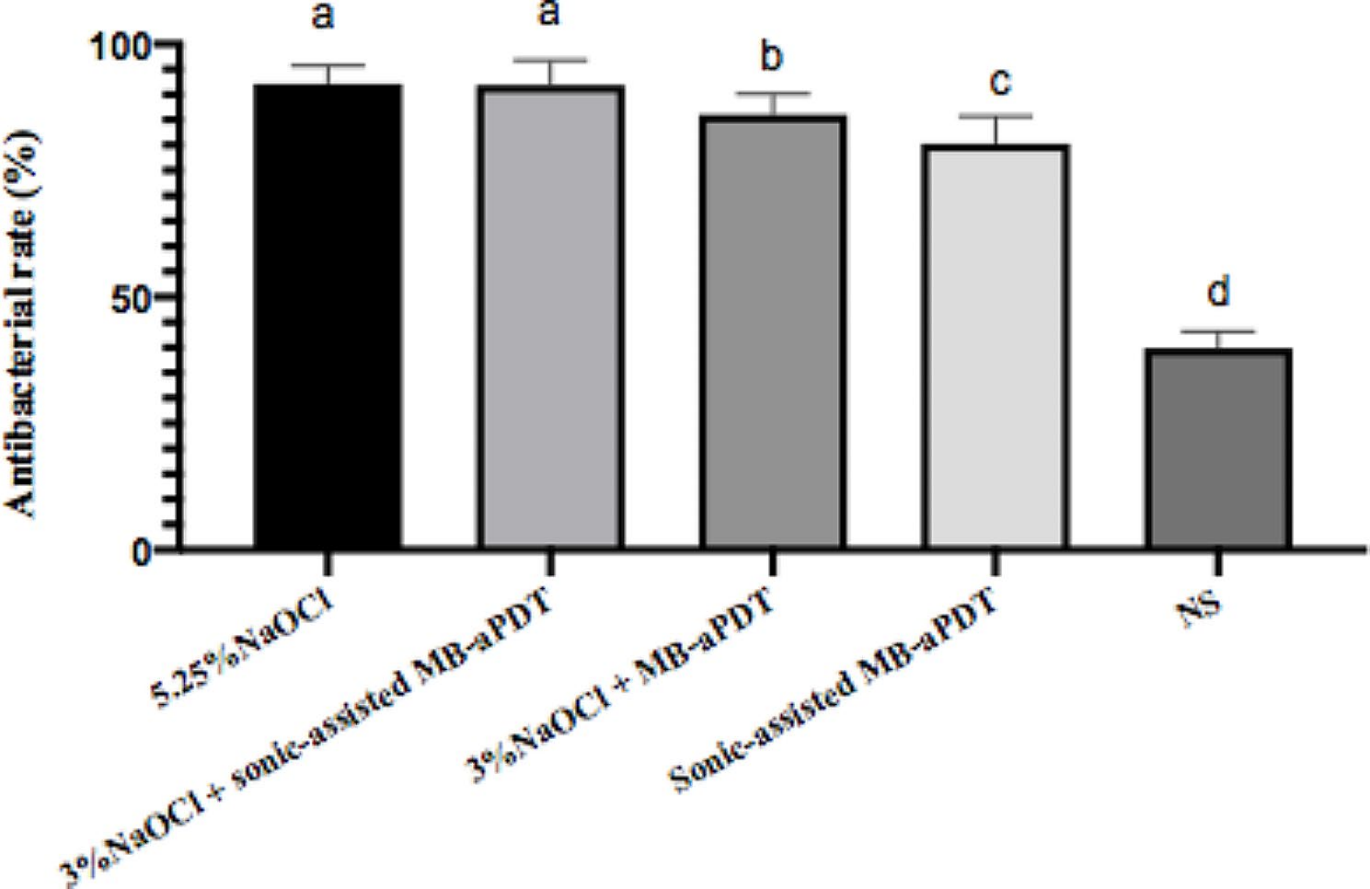
The antibacterial rate of different groups against *E. faecalis* in lateral canals. The different letters represented statistical differences between groups (*P* < 0.05)

Figure 3 presents the findings of the study. The positive control group, treated with 5.25% NaOCl, and the group treated with 3% NaOCl + sonic-assisted MB-aPDT exhibited the highest antimicrobial outcomes, with antimicrobial rates of 92.15% and 91.86%, respectively. However, no statistically significant difference between these two groups was observed (P > 0.05). The antimicrobial efficacy of 3% NaOCl + MB-aPDT (86.06%) was significantly higher than that of the sole sonic-assisted MB-aPDT group (80.32%) (P < 0.05). In contrast, the negative control group (NS) exhibited the lowest antibacterial rate of 39.86%.

### SEM analysis

**Fig. 4:**
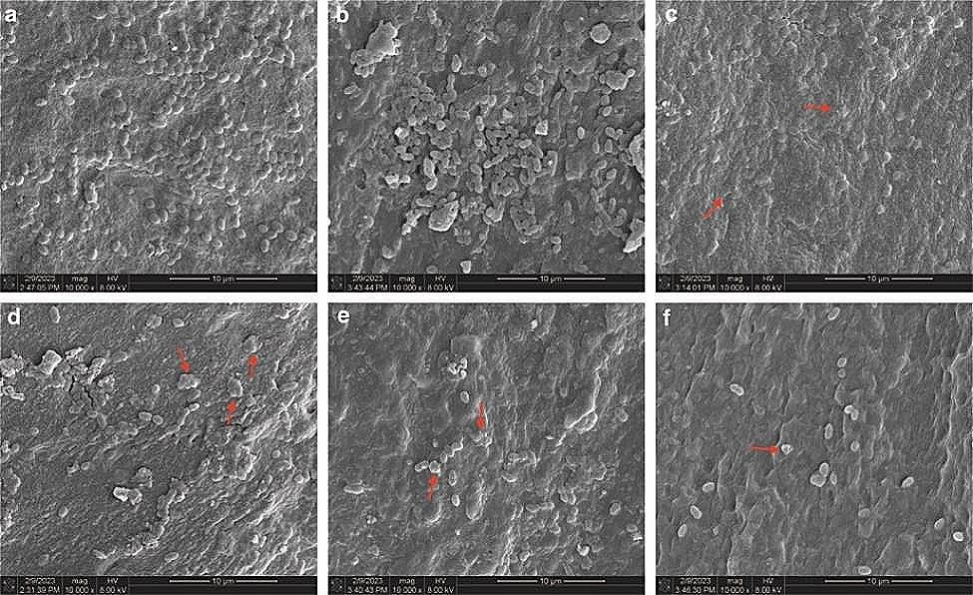
Representative images of the SEM examination after different treatments. (**a**) Untreated group; (**b**) NS; (**c**) Sonic-assisted MB-aPDT; (**d**) 3% NaOCl + MB-aPDT; (**e**) 3% NaOCl + sonic-assisted MB-aPDT; (**f**) 5.25% NaOCl

Different treatments resulted in observed morphological changes of *E. faecalis* in lateral canals, as depicted in Fig. 4. Unlike the typical form of *E. faecalis* (depicted in Fig. 4a), all experimental groups, excluding the negative control group, exhibited alterations in bacterial morphology, as highlighted by the red arrows.

## Discussion

Lateral canals are small gaps that form during the development of the epithelial root sheath, which connect the root canal to the periodontal and provide a pathway for bacterial infection between the pulp and periodontal tissues [5]. The concealed anatomical location and narrow space of the lateral canals renders it difficult to eliminate, ultimately leading to the ineffectiveness of root canal therapy. Hence, eliminating bacteria in the lateral canals is crucial for enhancing the efficacy of root canal therapy and improving success rates.

Previous research has suggested diverse approaches, such as employing naturally extracted teeth and transparent resin block models, for simulating lateral canals [32–35]. Transparent resin block models offer numerous benefits, including visual clarity, ease of acquisition, and standardization. However, the chemical composition of these models deviates from that of natural teeth, which may reduce the adherence of bacterial biofilms to their surfaces [36]. In contrast, naturally extracted teeth possess abundant collagen fibers within the dentin, which facilitate bacterial adhesion and the formation of stable bacterial biofilms. Therefore, this study chose naturally extracted teeth as the experimental specimens. It is worth noting that previous research has reported a wide range of lateral canal diameters, ranging from 16.7 to 238.44 μm [37], and from 50 to 150 μm [38]. Considering these observations, a 100 μm diameter was selected for the artificial lateral canals, achieved using a 10# K-file, as it closely resembles the dimensions of natural lateral canals.

In this study, a series of centrifugation techniques were employed to introduce bacteria into the lateral canals, thereby ensuring a uniform depth and density of bacterial colonization and facilitating a more expedient establishment of the infection model. In contrast to the centrifugation method proposed by Andrade et al. [31], the duration of bacterial incubation in our study was set at two weeks. This decision was made to allow the bacteria sufficient time to recover from any potential damage caused by high-speed centrifugation and to develop into mature biofilms. SEM analysis in this study revealed that *E. faecalis* was able to withstand potential damage resulting from high-speed centrifugation after a two-week incubation period. This phenomenon could be attributed to the bacteria’s ability to tolerate adverse environments [39].

Our current study demonstrated that sonic-assisted could help to enhance the antibacterial efficacy of MB-aPDT, and the combination of 3% NaOCl and sonic-assisted MB-aPDT achieved a synergistic promotive effect which exhibited an antibacterial effect comparable to that of 5.25% NaOCl. It is worthwhile to notify that the use of NaOCl solution for root canal irrigation can pose a risk to host cells as the concentration of the solution increases [15, 38]. Conversely, previous studies have shown that aPDT has minimal toxicity to host cells [40, 41]. The concentration of photosensitizer used in this study was 0.01%. The safety of this concentration has been fully confirmed in previous studies [42]. Additionally, this concentration of MB-aPDT is currently being clinically used [43, 44]. Moreover, for the combined treatment group in this experiment, we selected 3% NaOCl for irrigation, as this concentration of the solution has been demonstrated to possess effective antimicrobial properties while maintaining its safety [45, 46]. Therefore, the utilization of 3% NaOCl in conjunction with sonic-assisted MB-aPDT proposes a novel approach for managing lateral canal infections, providing a comparable antibacterial impact as the 5.25% NaOCl solution while reducing the potential risk of tissue damage.

The present study observed that all experimental groups, except the negative control group, exhibited bacterial morphological alterations. This observation suggests a potential association with the distinct antibacterial mechanisms employed by each protocol. The antibacterial mechanism of NaOCl is mainly through its hydrolysis to form hypochlorous acid, which further decomposes to form new ecological oxygen [47]. This new ecological oxygen possesses strong oxidative properties that can denature bacterial protein and lead to bacterial death [48]. On the other hand, in the experimental groups that involved MB-aPDT, it is speculated that ROS generated during the aPDT process may disrupt specific substructures within bacterial cells or interfere with bacterial metabolic functions, thereby exerting its antibacterial effect [49].

The efficacy of the 3% NaOCl + sonic-assisted MB-aPDT treatment in eradicating *E. faecalis* in the lateral canals was found to be effective, although complete elimination of the bacteria was not achieved. There are several potential reasons for this. Firstly, the presence of a mature biofilm structure of *E. faecalis* in the lateral canals may impede the penetration of photosensitizers, thereby compromising the antibacterial effectiveness. Secondly, the low-oxygen environment within the lateral canals may lead to an imbalanced ratio of ROS output to input, which can negatively affect the antibacterial activity. Lastly, it is important to note that photosensitizers used in photodynamic therapy may undergo photobleaching, a common phenomenon [50]. The continuous reduction of photosensitizer molecules during the treatment process has the potential to diminish its antibacterial efficacy. In summary, gaining a comprehensive understanding of the bacterial microenvironment in the lateral canals and the photobleaching process of the photosensitizer could enhance the efficiency of MB-aPDT in disinfecting infected root canals.

The results of this research indicate that the utilization of 3% NaOCl in combination with sonic-assisted MB-aPDT is more effective in eradicating *E. faecalis* in lateral canals compared to sonic-assisted MB-aPDT alone. Therefore, sonic-assisted MB-aPDT can be considered as an adjunctive tool for root canal disinfection. Additionally, the results suggest that the combination of 3% NaOCl with sonic-assisted MB-aPDT is more effective than the combination of 3% NaOCl with MB-aPDT alone, indicating that sonic-assisted techniques can enhance the antibacterial properties of MB-aPDT. This result may be attributed to the ability of sonic treatment to improve the permeability of photosensitizers in aPDT [29].

## Conclusion

It is reasonable to conclude that the combination of 3% NaOCl and sonic-assisted MB-aPDT can achieve comparable antibacterial activity to 5.25% NaOCl. However, this experiment only focused on evaluating the antibacterial activity in straight and mature single-rooted premolars, leaving uncertainty regarding the antibacterial effects in complex root canals. Therefore, investigating the antibacterial effects of sonic-assisted MB-aPDT in curved molar root canals will be a focus of our future work.

## Data Availability

The datasets used and/or analyzed during the current study are available from the corresponding author on reasonable request.

## Abbreviations

aPDT: Antibacterial Photodynamic Therapy
ATCC: America Type Culture Collection
BHI: Brain Heart Infusion
CCM: Colony Counting Method
CFU: Colony Forming Unit
EDTA: Ethylene Diamine Tetraacetic Acid
*E. faecalis*: *Enterococcus faecalis*
MB: Methylene Blue
NaOCl: Sodium Hypochlorite
NS: Normal Saline
PBS: Phosphate Buffered Saline
SEM: Scanning Electron Microscope
